# Current Progress in Tonoplast Proteomics Reveals Insights into the Function of the Large Central Vacuole

**DOI:** 10.3389/fpls.2013.00034

**Published:** 2013-03-01

**Authors:** Oliver Trentmann, Ilka Haferkamp

**Affiliations:** ^1^Pflanzenphysiologie, Technische Universität KaiserslauternKaiserslautern, Germany

**Keywords:** vacuole, tonoplast, proteomics, phosphoproteome studies, comparative proteome studies

## Abstract

Vacuoles of plants fulfill various biologically important functions, like turgor generation and maintenance, detoxification, solute sequestration, or protein storage. Different types of plant vacuoles (lytic versus protein storage) are characterized by different functional properties apparently caused by a different composition/abundance and regulation of transport proteins in the surrounding membrane, the tonoplast. Proteome analyses allow the identification of vacuolar proteins and provide an informative basis for assigning observed transport processes to specific carriers or channels. This review summarizes techniques required for vacuolar proteome analyses, like e.g., isolation of the large central vacuole or tonoplast membrane purification. Moreover, an overview about diverse published vacuolar proteome studies is provided. It becomes evident that qualitative proteomes from different plant species represent just the tip of the iceberg. During the past few years, mass spectrometry achieved immense improvement concerning its accuracy, sensitivity, and application. As a consequence, modern tonoplast proteome approaches are suited for detecting alterations in membrane protein abundance in response to changing environmental/physiological conditions and help to clarify the regulation of tonoplast transport processes.

## Introduction

Plant vacuoles can be distinguished by their size, specific metabolic properties, state of maturation, etc. Moreover, their number and physiological role can undergo dramatic changes during cell cycle and development (Segui-Simarro and Staehelin, 2006; Oda et al., 2009). The two main functional types of vacuoles are lytic vacuoles and protein storage vacuoles. The large central lytic vacuole can account for up to 90% of the total cell volume. It is required for turgor maintenance and represents the main storage site for minerals and nutrients (Marty, 1999). Moreover, it is involved in cellular signaling and detoxification. Reproductive tissues, such as seeds, generally contain a large number of protein storage vacuoles. These vacuoles are smaller in size and act as an important nutrient reservoir for the developing embryo. Selective transport of ions and molecules across the tonoplast determines the function of the different types of vacuoles (Jauh et al., 1999; Marty, 1999; Maeshima, 2001). Diverse tonoplast transport properties were deduced from biochemical measurements on isolated intact (mainly lytic) vacuoles or tonoplast vesicles from different plant species (Martinoia et al., 2007). However, the molecular nature of many proteins involved in the corresponding transport processes is still not clarified. Specific proteome analyses aim to identify soluble proteins of the vacuolar lumen, tonoplast intrinsic, or associated proteins and provide important insights into the regulation of vacuolar protein abundance and post-translational modification.

## Vacuole Preparation and Tonoplast Membrane Enrichment

The gain of sufficient amounts of vacuoles or tonoplast membranes that exhibit adequate purity is an essential prerequisite for conclusive proteome analyses. However, preparation of intact vacuoles, particularly of large central lytic vacuoles, is hampered by their large size because shear forces can disrupt the organellar integrity. In the early 1960s, scientists succeeded in the enrichment of intact vacuoles from different plant species. In the first documented study, Cocking isolated vacuoles by osmotic lysis from protoplasts that were generated from young tomato root tip tissue (Cocking, 1960). Fifteen years later, Wagner and Siegelman established large-scale isolation of vacuoles from protoplasts of different plant organs, including mature leaves, flower petals, stems, filaments, styles, and young fruits from various plant species, e.g., *Hippeastrum*, *Tulipa*, *Ipomea*, or *Pisum* (Wagner and Siegelman, 1975). However, protoplast generation by fungal cellulase treatment is apparently inapplicable for vacuole isolation from robust tissues (Leigh and Branton, 1976). Leigh and Branton demonstrated that successful isolation of vacuoles from the storage root of *Beta vulgaris* is possible after gentle mechanical disruption of the tissue (tissue slicing) (Leigh and Branton, 1976). On the basis of the three given fundamental studies (Cocking, 1960; Wagner and Siegelman, 1975; Leigh and Branton, 1976) vacuole preparation has been optimized continuously, particularly with respect to purity and yield. Physiological analyses on correspondingly isolated vacuoles allowed identifying acidic hydrolase activity (Boller and Kende, 1979) as well as transport and luminal accumulation of different metabolites (carboxylates, sugars, amino acids, nitrate, ions) and anthocyanines (Wagner, 1979; Martinoia et al., 1981; Hedrich et al., 1986; Hedrich and Kurkdjian, 1988; Rentsch and Martinoia, 1991).

Because *Arabidopsis thaliana* is one of the most important model systems in plant physiology the knowledge of protocols for isolation of intact vacuoles from this plant species is of high importance for plant science. Vacuole isolation by osmotic lysis of protoplasts from *Arabidopsis* leaves or cell cultures was used for, e.g., subcellular localization of ubiquitinated proteins (Beers et al., 1992), for analysis of flavone glucoside uptake (Frangne et al., 2002), or functional investigation of the cation/H^+^ exchanger *At*NHX1 (Apse et al., 1999, 2003), the monosaccharide carrier *At*TMT (Wormit et al., 2006), or the malate carrier *At*TDT (Emmerlich et al., 2003) as well as for proteome studies (Carter et al., 2004; Shimaoka et al., 2004).

**Figure 1:**
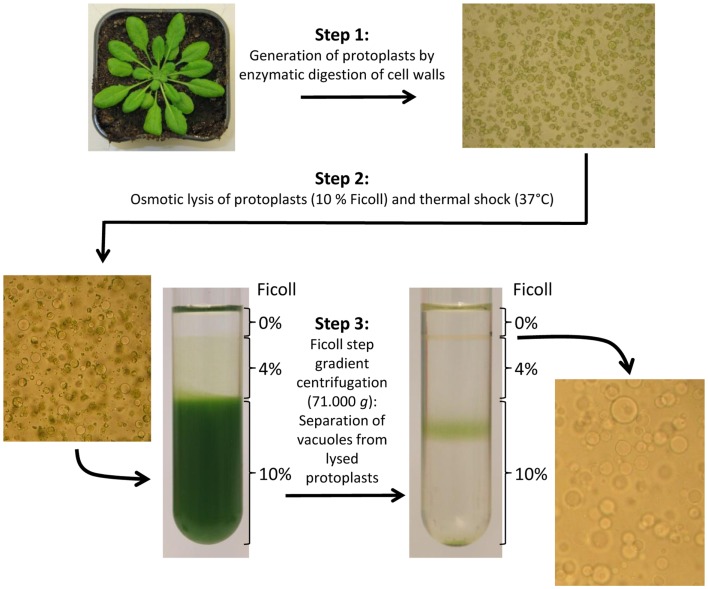
**Preparation of intact *Arabidopsis thaliana* vacuoles by generation of mesophyll cell protoplasts, osmotic/thermal lysis of protoplasts, and vacuole purification by use of a Ficoll step gradient (according to Robert et al., 2007)**. Microscopic pictures; 100-fold magnified.

In 2007, a very detailed description of intact vacuole isolation from *Arabidopsis* leaves including a trouble shooting guide was published (Robert et al., 2007). This isolation procedure results in concentrated vacuoles of comparably high purity and hence not only represents a suitable basis for proteome analyzes (Carter et al., 2004) but also for the determination of luminal solute contents (Klaumann et al., 2011; Schulze et al., 2012). The complete preparation procedure takes about 6–8 h (starting from harvest of leaf material) and generally comprises three major work steps: 1. Generation of leaf cell protoplasts. 2. Lysis of protoplasts for liberation of intact vacuoles. 3. Enrichment of vacuoles by ultracentrifugation in a three-phase Ficoll gradient. Figure 1 presents an overview about this important method and the three working stages. Moreover, tonoplast membranes can be easily collected from the vacuole fraction by ultracentrifugation (>100.000 × *g*) (Carter et al., 2004; Schulze et al., 2012). Practical experience in isolation of vacuoles from *Arabidopsis* led us to the conclusion that leaves of 4–6 weeks old plants grown under short day conditions are ideal to obtain high yields of vacuoles. Additionally, it is advisable to harvest plant tissue approximately 2–4 h before onset of light (unpublished data).

Vacuole isolation from protoplasts might be considered as problematic because cell wall lysis takes about 2–4 h and hence might represent a stress situation for the cells and generated protoplasts. Therefore, it is imaginable that some analyses are affected by this putative stress situation. Particularly, comparative studies, including quantitative proteome investigations of vacuoles isolated from plants after application of abiotic or biotic stress factors, have to be critically evaluated. Application of suitable controls performed in parallel helps to identify “artifacts” caused by the isolation method. Metabolite measurements of vacuoles isolated from cold acclimated and control plants demonstrated a significant increase in glucose and fructose concentrations solely in those vacuoles that were isolated from the plants incubated at 4°C (Schulze et al., 2012). Moreover, cold acclimation was shown to be accompanied by a general cellular increase of soluble sugars (Schulze et al., 2012). This demonstrates that vacuole isolation from protoplasts is suitable to detect metabolic alterations induced by the applied environmental factors.

Enrichment of tonoplast vesicles from crude membrane preparations represents an alternative procedure for the determination of vacuolar transport functions. Tonoplast vesicles from homogenized plant material fuse to small vesicles of low-density that can be separated from microsomes with a different membrane composition by density gradient centrifugation (Mettler and Leonard, 1979; Dupont et al., 1982; Churchill and Sze, 1983) or Free Flow Electrophoresis (Barkla et al., 2007, 2009). This method was applied for, e.g., characterization of proton transport and ATPase activity in various plants (Dupont et al., 1982; Churchill and Sze, 1983; Poole et al., 1984) or of Ca/H^+^ antiport (Schumaker and Sze, 1985) and pyrophosphatase activity (Wang et al., 1986) in oat root tonoplasts. Tonoplast vesicles were also used for qualitative (Szponarski et al., 2004) and comparative proteome analyses (Barkla et al., 2009).

## Qualitative Vacuole/Tonoplast Proteome Studies of *Arabidopsis thaliana*

Although physiological and biochemical studies provided important insights into the role of the large central vacuole comprehensive information about the tonoplast protein composition was missing until the millenium. In 2004, the first proteome study by Szponarski et al. (2004) resulted in the detection of 70 proteins. Subsequent analyses by Shimaoka et al. (2004), Carter et al. (2004), and Jaquinod et al. (2007) identified 163, 402, and 650 proteins, respectively. The increased number of proteins that were identified in the different studies suggests that the used plant material, the applied vacuole isolation technique, sample preparation, and/or protein detection method influence the quantity of vacuolar proteome data (Table 1). Either suspension cultures of cells grown in permanent light (Szponarski et al., 2004; Jaquinod et al., 2007) or in permanent darkness (Shimaoka et al., 2004) or rosette leaves of plants grown on soil under short day conditions were used (Carter et al., 2004). Shimaoka et al., Carter et al., and Jaquinod et al. isolated vacuoles by osmotic lysis from protoplasts whereas Szponarski et al. prepared tonoplast vesicles from crude membrane fractions. Enrichment and purification of vacuoles or tonoplast vesicles was achieved by centrifugation using different types of density gradient media, either sucrose (Szponarski et al., 2004), Percoll (Shimaoka et al., 2004), or Ficoll (Carter et al., 2004; Jaquinod et al., 2007). Different samples of tonoplast proteins were analyzed by MALDI-TOF-MS (Szponarski et al., 2004) or LC-MS/MS (Shimaoka et al., 2004). However, most proteins were identified when differently treated samples of total vacuoles, tonoplast membranes (Carter et al., 2004; Jaquinod et al., 2007), and soluble proteins (Jaquinod et al., 2007) were subject to LC-MS/MS. Therefore, we conclude that not the chosen plant material (leaf tissue versus suspension culture cells) but rather the vacuole/tonoplast isolation procedure, the quality of the prepared samples and the applied detection method influence the quantity of identified proteins.

**Table 1:** **Published vacuolar proteome studies from 2004 to 2007**.

Author	Plant material used	Method	Purity control	Sample preparation	Mass spectrometer	Proteins identified
Szponarski et al. (2004)	*Arabidopsis thaliana* suspension cells – permanent light	Tonoplast membranes separated from total microsomal membranes by sucrose step gradient	–	Solubilization of membrane proteins using a mild detergent Gel filtration Anion exchange chromatography SDS PAGE In-gel digestion	BiFlexIII-MALDI-TOF Bruker Daltonics, Germany	70
Shimaoka et al. (2004)	*Arabidopsis thaliana* suspension cells – w/o light	Generation of protoplasts lysis of protoplasts separation of vacuoles using a Percoll step gradient	Western-blot/antibodies used: V-type H^+^ATPase Luminal binding protein BIP Plasma membrane H^+^-ATPase determination of marker enzyme activities	Purification of tonoplasts by ultracentrifugation Separation of tonoplast integral and peripheral proteins by SDS PAGE In-gel digestion	Q-TOF Ultima Waters Co., USA	163
Carter et al. (2004)	*Arabidopsis thaliana* leaves from 35 days old plants	Generation of protoplasts lysis of protoplasts separation of vacuoles using a Ficoll step gradient	Western-blot/antibodies used: Heat shock protein 93 Luminal binding protein BIP Cyclooxygenase-2 COX2 Syntaxin of plants 41 SYP41 Vacuolar endogenous protein ALEU GTPase SAR1 microscopic inspection of purified vacuoles	In-liquid digestion of total vacuolar proteins Separation of total vacuolar proteins by SDS PAGE and in-gel-digest Separation of tonoplast proteins by SDS PAGE and in-gel digestion	Q-TOF API US Waters Co., USA	402
Endler et al. (2006)	*Hordeum vulgare* (Barley) leaves from 8 days old plants	Generation of protoplasts mechanical lysis separation of vacuoles using a mixed Percoll/Ficoll step gradient	Western-blot/antibodies used: Tonoplast intrinsic protein TIP1-like Chloroplast ATP-synthase α subunit Luminal binding protein BIP Alternative oxidase AOX Plasma membrane intrinsic protein PIP determination of marker enzyme activities microscopic inspection of purified vacuoles	Separation of tonoplast proteins by SDS PAGE and in-gel digestion	LCQ Deca XP ion trap Thermo Finnigan Thermo, USA	101
Jaquinod et al. (2007)	*Arabidopsis thaliana* suspension cells – permanent light	Generation of protoplasts lysis of protoplasts separation of vacuoles using a Ficoll step gradient	Western-blot/antibodies used: Outer envelope protein OEP21 Light harvesting complex LHCb Translocase outer mitochondrial membrane protein 40 Plasma membrane H^+^-ATPase Anti -HDEL-domain Tonoplast intrinsic protein TIP3;1 Tonoplast intrinsic protein TIP1	Separation of total vacuolar proteins by SDS PAGE and in-gel digestion In-gel or in-liquid digestion in of tonoplast membranes Separation of soluble fraction representing the vacuolar sap by SDS PAGE and in-gel digestion	Q-TOF Ultima Waters Co., USA	650
Schmidt et al. (2007)	*Brassica oleracea* (Cauliflower) buds obtained at commercial outlet	Slicing of buds separation of intact vacuoles using a nycodenz step gradient	Western-blot/antibodies used: Tonoplast intrinsic protein TIP1 Chloroplast ATP-synthase α subunit Luminal binding protein BIP Plasma membrane aquaporin PAQ1 determination of marker enzyme activities microscopic inspection of purified vacuoles	Separation of peripheral and integral tonoplast proteins by SDS PAGE In-gel digestion	LCQ Deca XP ion trap Thermo Finnigan Thermo, USA	316

Investigation of the *Arabidopsis* proteome helped to decipher the basic membrane protein composition of the large central vacuole from *Arabidopsis thaliana*. Prominent vacuolar proteins, like V-type ATPase subunits, pyrophosphatases, glycosidases, and tonoplast intrinsic proteins, as well as proteins involved in stress response, membrane remodeling, protein degradation, solute transport, or cytoskeleton anchoring and also numerous proteins of so far unknown function were identified (Carter et al., 2004; Shimaoka et al., 2004; Szponarski et al., 2004; Jaquinod et al., 2007). The different protein groups reflect important physiological functions of the vacuole, such as storage of metabolites (sugars, amino acids, ions, metal ions, including heavy metals), sequestration of toxic substances (xenobiotics), degradation of various cytosolic compounds, turgor maintenance, and stress response. The high abundance of pyrophosphatases and ATPase subunits demonstrates that proton pumps constitute a major part of tonoplast proteins. The generated proton gradient drives secondarily active transport processes and acidification of the vacuolar lumen supports luminal degradation processes.

Vacuolar proteome analyses provide an important basis for the functional analyses of proteins. For example, the characterization of the copper transporter COPT5 (Klaumann et al., 2011), the molybdate transporter MOT2 (Gasber et al., 2011), and the nucleoside transporter ENT1 (Bernard et al., 2011) was initiated by vacuolar proteome data.

## Qualitative Vacuole/Tonoplast Proteome Studies of Barley and Cauliflower

Even though *Arabidopsis* represents an important model organism in plant science it is not representative for all other plants. Particularly, plants distantly related to *Arabidopsis* are often characterized by a different physiology and metabolic regulation. Accordingly, it is interesting to discover differences, similarities or identities in the protein composition of organelles and membranes also in other plants than *Arabidopsis*.

Endler et al. (2006) investigated the proteome of vacuoles isolated from *Hordeum vulgare* mesophyll cells. Barley was cultivated on soil under long day conditions (16 h light) for 8 days. Cell wall digestion and generation of protoplasts was conducted as described in previous studies (Carter et al., 2004; Shimaoka et al., 2004), however disruption of protoplasts and release of intact vacuoles was not induced by osmotic shock but by mechanical force (shearing by pressing through a syringe). Vacuoles were purified and separated from residual protoplast debris by Percoll/Ficoll step gradient centrifugation. LC-MS/MS allowed the detection of 101 proteins, including 11 of 12 V-type ATPase subunits. The identification of the sucrose transporter *Hv*SUT2 in the vacuolar proteome was interesting because the molecular nature of carriers mediating the well-known sucrose transport across the tonoplast (Kaiser and Heber, 1984) was not known. GFP-based localization studies confirmed that *Hv*SUT2 and its closest homolog from *Arabidopsis* *At*SUT4 reside in the tonoplast (Endler et al., 2006).

Schmidt et al. analyzed the vacuolar protein composition of young meristematic cauliflower cells (Schmidt et al., 2007). Because cauliflower buds represent a rather robust tissue, vacuoles were not isolated from protoplasts but from crude extracts by mechanical disruption of the tissue, filtration, and centrifugation. Vacuoles were separated from other organelles by the help of a discontinuous Nycodenz-based gradient and ultracentrifugation and subsequently disrupted by hypotonic lysis. Tonoplasts were further concentrated by ultracentrifugation. Integral and peripheral membrane proteins were separated and in total 316 proteins were identified by LC-MS/MS (Schmidt et al., 2007). GFP-localization studies in *Arabidopsis thaliana* protoplasts and onion epidermal cells verified vacuolar targeting of five novel identified proteins. The obtained proteome data were compared to results of vacuolar *Arabidopsis* proteome studies (Carter et al., 2004; Shimaoka et al., 2004; Szponarski et al., 2004).

In vacuoles of cauliflower and *Arabidopsis*, more than 800 non-redundant proteins were identified. These proteins are either located in the vacuolar sap or represent tonoplast intrinsic or peripheral proteins.

## The Problem of Contamination

The aim of vacuolar proteome studies is to determine the protein composition of this organelle. The purity of the preparation, more precisely the presence of non-vacuolar, contaminating proteins, represents one of the major problems affecting the validity of proteome data.

The quality of vacuolar and tonoplast preparations can be analyzed by measuring specific enzyme activities, by Western-blotting using antibodies raised against organellar marker proteins, and/or by light microscopy.

Activity assays of enzymes located in various organelles/membranes were performed and documented in the qualitative proteome studies by Shimaoka et al. (2004) and Endler et al. (2006). In these studies as well as in the study conducted by Jaquinod et al. vacuolar enrichment during the preparation procedure was monitored. All proteome analyses (except that of Szponarski et al.) investigated the quality and purity of the samples by immunodetection using antibodies raised against different typical marker proteins of the tonoplast, the plasma membrane, ER, and Golgi-network, chloroplasts, and mitochondria (for details see Table 1).

Microscopic analyses of enriched vacuoles were documented in only few proteome studies (Carter et al., 2004; Endler et al., 2006; Schmidt et al., 2007). The informative value of a microscopic investigation of isolated vacuoles is rather limited because solely contaminations of larger dimensions are detectable. However, it is easy applicable and allows immediate (on-line) determination of vacuole enrichment and purity and hence is a suitable method, particularly during establishment and optimization of the preparation protocol. Non-lysed protoplasts and chloroplasts still present in the sample can be identified easily. Samples that contain contaminations already detectable by light microscopy are generally unsuited material for significant proteome studies.

Until now it seems to be technically not feasible to generate an organelle fraction totally free from other cellular components and increased sensitivity of mass spectrometry allows detecting even minimal impurities. Critical examination of the mass spectrometry results helps to address the problem of contamination and to evaluate the validity of vacuolar proteome data. The identification of a high number of proteins with proven vacuolar localization and a low recovery/abundance of contaminating proteins indicates successful enrichment of vacuoles and/or tonoplasts. It is necessary to keep in mind that detection of few non-vacuolar proteins might also result from weak sensitivity of the analysis. Accordingly, not only the protein type (vacuolar versus other compartment) but also the number of detected peptides (of a single protein) as well as protein coverage have to be considered.

The degree of protein sequence coverage reflects the general sensitivity of the detection method and therefore, the identified peptides should cover a large area of the corresponding protein amino acid sequence. The V-type ATPase is constituted of 12 subunits and the *Arabidopsis* genome encodes 28 proteins proven or at least predicted to represent V-type ATPase subunits, including different subunit isoforms (Sze et al., 2002). Most ATPase subunit proteins (19) were identified by Jaquinod and coworkers and these subunits included all 12 proteins required to build the complete complex.

The number of detected peptides reflects the abundance of proteins in the sample. Most peptides should represent prevalent vacuolar proteins, like V-type ATPase and pyrophosphatase, whereas fewer peptides of vacuolar proteins of lower abundance are expected. Although activity measurements clearly demonstrated the presence of several functionally different channel proteins in the tonoplast corresponding candidate proteins were rarely detected in vacuolar/tonoplast proteomes (Ca^2+^ activated channel TPC1; Carter et al., 2004; Szponarski et al., 2004; Jaquinod et al., 2007; and the tandem pore K^+^ channel TPK1/KCO1; Schulze et al., 2012). The absence of certain electrophysiologically identified channel proteins in proteome analyses might be explained by a very low abundance. This demonstrates the particular potential of electrophysiological analyses which even allow the detection and investigation of a single channel protein (by e.g., patch clamp technique).

Low amounts of the ER luminal binding protein (Carter et al., 2004) and of HDEL peptides (Jaquinod et al., 2007) were detected in some of the purified vacuole samples. However, it is not unequivocally clarified whether certain ER or trans-Golgi-network derived proteins represent vacuolar components or contaminations. This is because protein and lipid trafficking to the vacuole is largely mediated via the secretory pathway (Marty, 1999).

Although mitochondria and plastids were not identified as relevant contaminants (by Western-blotting), almost all qualitative vacuolar proteome analyses contained well-known plastidial and mitochondrial proteins, like the small subunit of ribulose-1,5-bisphosphate carboxylase/oxygenase, chlorophyll binding proteins of the light harvesting complex, and the mitochondrial ATP-synthase or the ADP/ATP carrier (Carter et al., 2004; Shimaoka et al., 2004; Endler et al., 2006; Jaquinod et al., 2007; Schmidt et al., 2007). It is very likely that these contaminating proteins are often detected because they are among the most abundant proteins of mitochondria or plastids. The overall quality of the obtained vacuolar proteomes is not generally downgraded by detecting proteins that are highly abundant in mitochondria or chloroplasts. Their identification rather demonstrates the sensitivity of mass spectrometry compared to, e.g., Western-blot analyzes or enzyme activity tests. In this context it has to be considered that the quality, sensitivity, and specificity of the used antibody and immunodetection method as well as the nature and abundance of the investigated protein are important factors influencing the significance of the result.

Increasing knowledge about the subcellular localization of more and more proteins and the establishment of sophisticated proteome processing databases will further enhance and support evaluation of vacuolar proteome results.

## Phosphoproteome Studies

One major function of the large central vacuole is the adjustment of cytosolic metabolite and ion levels by specific uptake or release of solutes in accordance to cellular demands. Various carriers and channel proteins in the tonoplast are involved in this process. To fulfill the required metabolic function the amounts of corresponding tonoplast proteins, their activities or biochemical properties have to be modified and tightly regulated. Changes in protein levels and composition are generally caused by alterations in gene expression and/or protein synthesis whereas post-translational modifications rather affect the biochemical properties of the respective proteins.

Phosphorylation is a biologically important and prevalent post-translational modification of soluble and membrane proteins. Addition of a phosphate group is catalyzed by kinases and alters the biochemical properties, like, e.g., activity or substrate affinity, of the target protein. Indications for a possible interaction of kinases and vacuolar proteins were obtained from analyses of SOS2 mutant plants. The protein kinase SOS2 (also known as *At*SnRK3.11/CIPK24) is part of the salt-overlay-sensitive (SOS) pathway and was shown to activate the plasma membrane resident Na^+^/H^+^ antiporter SOS1 (also known as *At*NHX7) (Qiu et al., 2002). Interestingly, SOS2 is apparently also involved in vacuolar sodium transport regulation. Mutant plants lacking SOS2 exhibit reduced vacuolar Na^+^/H^+^ exchange whereas addition of activated SOS2 protein stimulates the corresponding transport (Qiu et al., 2004).

Mass spectrometry based phosphoproteome studies allow to identify whether tonoplast proteins are phosphorylated or not and whether this type of protein modification might play a virtual role in the regulation of vacuolar processes. In 2008, the first tonoplast phosphoproteome was published (Whiteman et al., 2008b). In this study, Whiteman et al. used *Arabidopsis* leaf material for the preparation of microsomes. Enrichment of tonoplast membrane containing vesicles from the microsomal fraction was achieved by discontinuous sucrose density gradient centrifugation. Tryptic digested phosphopeptides of the tonoplast were purified by use of GA^3+^-immobilized metal ion affinity chromatography (IMAC). At least one phosphopeptide was identified in 130 phosphorylated proteins. 58 phosphorylated proteins contained one or more transmembrane domains and hence were classified as membrane proteins. Twenty-eight of the 58 phosphorylated membrane proteins represent transport proteins specific for anions, sugars, potassium, sodium, or oligopeptides as well as ABC transporters (Whiteman et al., 2008b). Several of these phosphorylated transporters, like the tonoplast monosaccharide transporter *At*TMT1 or the tonoplast Na^+^/H^+^ exchanger NHX2, are proven components of the vacuole (Wormit et al., 2006; Bassil et al., 2011) whereas some carriers have been described to reside in the plasma membrane. The identification of a phosphorylated peptide assigned to NHX2 and the fact that SOS2 kinase regulates tonoplast Na^+^/H^+^ transport (Qiu et al., 2004) suggests that NHX2 might represent the vacuolar target protein of SOS2, however direct evidence is missing.

Interestingly, some (putative) protein kinases were identified in the tonoplast phosphoproteome (Whiteman et al., 2008b). In 2011, one of the identified kinases, the RAF-type MAPKKK-like kinase (also named Vik1, Ceserani et al., 2009), was analyzed with respect to a possible role in regulation of vacuolar sugar transporters. This kinase was shown to phosphorylate the inter-membrane loop region of the tonoplast monosaccharide transporter *At*TMT1. Import measurements revealed that presence of the recombinant RAF-type MAPKKK-like kinase stimulates glucose uptake into isolated vacuoles (Wingenter et al., 2011). Moreover, *Arabidopsis* lines lacking functional RAF-type MAPKKK-like kinase in many aspects pheno-copy mutants lacking tonoplast monosaccharide transport proteins (TMT’s) (Wingenter et al., 2011).

The identification of phosphorylated membrane proteins and particularly also of protein kinases in the tonoplast fraction, as well as vacuolar transport characteristics and metabolic phenotypes of kinase mutant plants (Qiu et al., 2004; Wingenter et al., 2011) suggest that kinase-mediated phosphorylation regulates vacuolar function.

Nearly simultaneously to the publication of the *Arabidopsis* phosphoproteome a further (phospho-)proteomic analysis of the plasma membrane and the tonoplast of *Oryza sativa* was published (Whiteman et al., 2008a). Whiteman and coworkers prepared tonoplast microsomes and microsomes containing plasma membrane proteins from shoot and root tissues of rice grown in a hydroponic growth system. In total 231 proteins were identified, including 94 membrane proteins. Sixty-one of these membrane proteins have been already functionally annotated as, e.g., primary H^+^ and Ca^2+^ pumps, ABC-type transporters and transporters for phosphate, potassium, sugars, or nitrogen. The detected membrane proteins and the number of corresponding peptides led the authors to the conclusion that certain carriers are nearly equally present in root and shoot tissues whereas other carriers, like, e.g., sugar transporters are of higher abundance in autotrophic plant organs.

Phosphorylated peptides of the different fractions were enriched by PHOS-Select™ iron affinity chromatography prior to the investigation of the phosphoproteome and finally 30 phosphopeptides were identified (Whiteman et al., 2008a). The observation that some *Arabidopsis* and rice orthologs exhibit a similar phosphorylation pattern (certain identical phosphorylated sites) suggests that this type of regulation is at least partially conserved between monocotyledones and dicotyledones. However, it is also imaginable that rice and maybe other monocot groups exhibit specific phosphorylation of proteins because several phosphopeptides were exclusively detected in rice (Whiteman et al., 2008a).

In 2009, Endler et al. published a phosphoproteome study of barley, a further agronomical important monocotyledonous model plant. Barley was cultivated and vacuoles were prepared according to the protocol used for investigation of the barley vacuolar proteome (Endler et al., 2009). Peptides of tonoplast proteins (from isolated vacuoles or tonoplast enriched microsomes) were purified by cation exchange chromatography and phosphopeptides were enriched by either IMAC (sepharose beads charged with FeCl_3_) or TiO_2_ affinity chromatography. Sixty-five identified phosphopeptides covered 27 tonoplast proteins of known biochemical function, such as two vacuolar proton pumps, aquaporins, calcium exchangers, Na^+^/H^+^ antiporters, and transport proteins for potassium, malate, sulfate, and sugar (Endler et al., 2009). Moreover, also calcium and chloride channels were shown to possess phosphorylated residues. Interestingly, a high number (12) of phosphorylation sites were determined within the large central loop of the barley hexose transporters *Hv*STP1/2, orthologs of *Arabidopsis* *At*TMT isoforms. The obtained results are largely in line with data of other tonoplast phosphoproteomes (Whiteman et al., 2008a,b) and suggest a certain degree of conservation but also species-specific adaptations in protein phosphorylation. However, we have to keep in mind that the phosphoproteome represents the current state of phosphorylation and this pattern might be affected by different growth conditions, biotic, or abiotic factors. Nevertheless, the phosphoproteome data clearly indicate that in various phylogenetically distantly related plant species transport processes across the tonoplast are regulated by phosphorylation.

In contrast to changes in gene expression and protein synthesis, post-translational protein modification (by phosphorylation) generally allows faster regulation. Therefore, the identification of phosphorylated tonoplast proteins is consistent with the physiologically required flexibility of vacuolar transport regulation. Continuative (large-scale) investigations will provide deeper insights into post-translational regulation of vacuolar transport and might discover further principles (additional post-translational modifications) controlling vacuolar function.

Comparative tonoplast phosphoproteome studies of plants that have been challenged with different stress stimuli, like cold, drought, nutrient deficiency, high environmental salt concentrations, heavy metals, or pathogens and of corresponding control plants will help to determine changes in phosphorylation pattern induced by biotic and abiotic factors. Although, diverse phosphorylated proteins have been identified, the regulatory impact of phosphorylation on the biochemical properties of the corresponding proteins is not clarified. Therefore, functional analysis of phosphorylated and de-phosphorylated forms of the identified proteins will decipher the functional consequences of these modifications. Moreover, it will be interesting to elucidate the way from signal perception and transduction to the modification of the final targets. The identification of phosphorylated residues in the monosaccharide transporters (TMT’s) provided the basis for the identification of the interacting RAF-type MAPKKK-like kinase. This kinase was shown to phosphorylate at least the *At*TMT1-loop and to stimulate sugar uptake into the vacuole (Wingenter et al., 2011).

## Comparative Proteome Studies

So far only few quantitative tonoplast/vacuolar proteome studies have been carried out.

Four different approaches are generally applied to distinguish between proteins from the different samples and to allow comparison of protein abundances (for detailed review see: Lilley and Dupree, 2006; Thelen and Peck, 2007). 2D-DIGE is a traditional key methodology in protein profiling studies. Different samples are labeled with different fluorescent dies and proteins are separated by two-dimensional gel electrophoresis. However, this technique is apparently unfavorable for the investigation of proteins with extreme pIs, high molecular weights, or of low abundant proteins. Moreover, hydrophobic proteins, such as membrane proteins, tend to precipitate during isoelectric focusing (Lilley and Dupree, 2006). Therefore, non-2D-gel based techniques might be of advance particularly for the analysis of membrane proteins. Prior to trypsin digestion either peptides/proteins can be labeled with chemical groups exhibiting the same molecular weight but disintegrate during mass spectrometry (tandem mass tags; **i**sobaric **t**ags for **r**elative and **a**bsolute **q**uantitation, iTRAQ), or specific amino acid residues can be labeled with stable isotopes (**i**sotope-**c**oded **p**rotein **l**abel, ICPL). Moreover, it is also possible to label proteins in living organisms by feeding stable isotopes (e.g., ^15^N salts supplied as nitrogen). This labeling procedure is not applicable for plants grown on soil and requires hydroponic plant cultivation or plant cell cultures. It is also possible to conduct a label-free quantification. An important advantage of a label-free strategy is the generally higher sensitivity (higher number of identified proteins). However, experimental replications and comparably demanding and extensive statistical analyses are necessary.

Besides its versatile functions the large central vacuole represents the cells main deposit for toxic compounds. A quantitative tonoplast proteome study of barley plants treated with increasing cadmium concentrations was performed to identify vacuolar proteins involved in the sequestration of this highly toxic metal (Schneider et al., 2009). Subsequent to Cd^2+^-treatment vacuoles were isolated from the leaves, tonoplast membranes were prepared and peptides were trypsin digested and labeled with commercially available iTRAQ reagent. Fifty-six tonoplast proteins were identified in this study. Treatment with low Cd^2+^ concentrations (20 μM) resulted in increased abundance of six proteins, the vacuolar H^+^ pyrophosphatase (gi|11527561), a TIP1-like protein (gi|520936), a putative natural resistance-associated macrophage protein (NRAMP) (gi|28865876), the vacuolar cation/proton exchanger CAX1a (gi|73917674), a multidrug-resistance(MRP)-like ABC transporter (gi|27368887), and an uncharacterized membrane protein (gi|115452029). In contrast to low Cd^2+^ concentrations, high Cd^2+^ concentrations (200 μM) apparently resulted in a more specific response because solely the abundance of the MRP-like protein was increased (Schneider et al., 2009). This study exemplifies that quantitative proteomes are suited to identify candidate proteins up-regulated by specific stress conditions. Investigation of mutant plants lacking or over-expressing the respective candidate proteins is required to pinpoint whether these proteins play a specific role in sequestration of Cd^2+^ and maybe also other toxic compounds.

*Mesembryanthemum crystallinum* is a facultative halophytic succulent commonly used for the investigation of plant responses to salt stress. Exposed to high environmental salinity or drought stress its metabolism switches from C3 to CAM (Crassulacean acid metabolism) photosynthesis (Osmond, 1978). *M*. *crystallinum* is an ideal candidate to study the vacuolar impact on salt tolerance and to identify salinity-induced modifications of the tonoplast protein composition. In 2009, Barkla et al. (2009) published a quantitative tonoplast proteome of plants osmotically challenged with 200 mM NaCl. In contrast to the previous proteome studies tonoplast membranes were purified by free flow zonal electrophoresis and the tonoplast proteins were subjected to two-dimensional differential in-gel electrophoresis (2D-DIGE) in combination with fluorescent labeling. 2D-DIGE might entail some disadvantageous, particularly with respect to the analysis of tonoplast proteins. Nevertheless, some proteins exhibiting altered abundance in response to salinity were detected. Two subunits of the V-type ATPase (VHA-d and VHA-B) and also two glycolytic enzymes, 2-phosphoglycerate dehydratase (enolase), and fructose-2-phosphate aldolase (aldolase), exhibited increased abundance. The identification of glycolytic enzymes in the context of vacuolar salt sequestration was unexpected. Barkla et al. conducted additional experiments to ascertain a possible interaction of the identified enolase and aldolase with tonoplast proteins. Chaotrophic treatment of the membranes, immunoprecipitation studies, and enzyme assays revealed that the corresponding glycolytic enzymes interact with subunit B of the V-type ATPase. Moreover, fructose-2-phosphate aldolase was shown to increase the affinity of V-type ATPase to its substrate ATP. Investigations of the *Arabidopsis* mutant *los2* confirmed an involvement of the identified enolase in salt stress metabolism.

A quite recent study focused on the role of the large central vacuole in adaptation to cold. Decreasing environmental temperatures initiate a process called cold acclimation (Schulze et al., 2012). Intensive physiological studies demonstrated that cold acclimation is associated with metabolic alterations. Particularly, cold-hardy species show immense accumulation of solutes, like proline and sugars (Wanner and Junttila, 1999; Stitt and Hurry, 2002). Schulze and coworkers conducted a label-free quantitative proteome analysis to investigate changes in tonoplast protein abundance in response to cold temperatures. To induce cold acclimation *Arabidopsis* plants were incubated at 4°C for several days. Intact vacuoles of cold acclimated and control plants were isolated according to the procedure described by Robert et al. (2007). Four biological replicates were utilized to statistically confirm changes in protein abundance. In total 778 *Arabidopsis* proteins were detected and quantified. About 64% of all identified peptides were affiliated to known vacuolar proteins. Eighteen tonoplast proteins showed significantly altered abundance upon cold acclimation. Thirteen proteins, including V-type pyrophosphatase as well as two integral subunits (VHA-d2 and VHA-c4) and one peripheral subunit (VHA-E1) of the V-type ATPase, exhibited higher abundance. Enzyme activity measurements were conducted to substantiate the involvement of V-type ATPase in cold acclimation. In fact, ATPase activity in tonoplast vesicles was considerably enhanced (at least fourfold) due to cold exposure. Therefore, the higher abundance of V-type ATPase subunits apparently results in a higher enzymatic activity. Cold acclimation resulted in higher abundance of nine solute transporters, including well-characterized tonoplast carriers, like the dicarboxylate transporter *At*TDT, the high affinity nitrate transporter *At*NRT2.7, and the zinc/H^+^ antiporter *At*MTP1/ZAT1 (Schulze et al., 2012). HPLC-based metabolite measurements revealed that cold acclimation leads to significantly higher vacuolar concentrations of malate and fumarate. These two dicarboxylates represent the main substrates of *At*TDT (Emmerlich et al., 2003) and hence their accumulation in vacuoles of cold-treated plants might be explained by the observed higher abundance of *At*TDT.

Determination of soluble sugar contents revealed that cold acclimation results in several-fold higher concentrations of sucrose, glucose and fructose, and that accumulation of the monosaccharides largely occurs in the vacuole. Interestingly, abundance of the main vacuolar monosaccharide transporters *At*TMT1 and *At*TMT2 (Wormit et al., 2006) remained unchanged. Phosphoproteome studies (Whiteman et al., 2008b; Endler et al., 2009) and a targeted investigation of phosphorylation sites in *At*TMT proteins revealed that cold acclimation stimulates phosphorylation of both, *At*TMT1 and *At*TMT2. Therefore, increased vacuolar accumulation of monosaccharides might result from higher activity of the TMT proteins caused by phosphorylation. Proteome data in combination with physiological studies for the first time clearly demonstrated that the vacuole is involved in the process of cold acclimation (Schulze et al., 2012).

## Conclusion and Outlook

Proteome studies considerably broadened the knowledge about the large central vacuole of plants. Qualitative proteome analyses identified various vacuolar and particularly, tonoplast proteins with increasing accuracy. Based on these important findings plant scientists managed to clarify the biochemical function and physiological relevance of several vacuolar proteins. Phosphoproteome data and physiological studies demonstrated that vacuolar proteins are modified by phosphorylation and that post-translational modification might represent an important mechanism allowing fast adaptation of vacuolar functions in accordance to the cellular demands. Comparative proteome studies elucidated the regulatory impact of abiotic stresses on vacuolar protein abundance. Sophisticated comparative proteome studies, might help to identify specific protein modifications induced by changing conditions. Investigation of tonoplast protein phosphorylation in *Arabidopsis* t-DNA-insertion lines lacking certain protein kinases might be a promising approach for the identification of signal pathways controlling vacuolar protein phosphorylation. A further challenge will be the large-scale investigation of the protein composition of non-lytic vacuoles, like protein storage vacuoles or pre-vacuolar vesicles. Comparison of the respective results might help to define differences between different types of vacuoles.

## Conflict of Interest Statement

The authors declare that the research was conducted in the absence of any commercial or financial relationships that could be construed as a potential conflict of interest.
